# Aluminium adjuvants and adverse events in sub-cutaneous allergy immunotherapy

**DOI:** 10.1186/1710-1492-10-4

**Published:** 2014-01-20

**Authors:** Christopher Exley

**Affiliations:** 1 The Birchall Centre, Lennard-Jones Laboratories, Keele University, Staffordshire, UK

**Keywords:** Aluminium adjuvant, Immunotherapy, Allergy, Adverse events

## Abstract

Sub-cutaneous immunotherapy is an effective treatment for allergy. It works by helping to modify or re-balance an individual’s immune response to allergens and its efficacy is greatly improved by the use of adjuvants, most commonly, aluminium hydroxide. Aluminium salts have been used in allergy therapy for many decades and are assumed to be safe with few established side-effects. This assumption belies their potency as adjuvants and their potential for biological reactivity both at injection sites and elsewhere in the body. There are very few data purporting to the safety of aluminium adjuvants in allergy immunotherapy and particularly so in relation to longer term health effects. There are, if only few, published reports of adverse events following allergy immunotherapy and aluminium adjuvants are the prime suspects in the majority of such incidents. Aluminium adjuvants are clearly capable of initiating unwanted side effects in recipients of immunotherapy and while there is as yet no evidence that such are commonplace it is complacent to consider aluminium salts as harmless constituents of allergy therapies. Future research should establish the safety of the use of aluminium adjuvants in sub-cutaneous allergy immunotherapy.

## Review

### Aluminium salts are the adjuvant of choice in immunotherapy

Aluminium-based adjuvants are the most commonly used adjuvant in sub-cutaneous allergy immunotherapy (SCIT) [1]. Approximately 75% of all such adjuvant-based therapies include an aluminium salt. In parallel with the use of aluminium-based adjuvants in vaccinations these adjuvants have been in use in allergy immunotherapies for over 80 years [2]. In allergy immunotherapy injections are sub-cutaneous and each injection includes between 0.1 and 1.25 mg of aluminium adjuvant. Allergy-specific immunotherapy can involve as many as 15 injections of aluminium adjuvant in a year and a treatment may be continued for 3 to 5 years or even longer, sometimes for the life-time of a patient [3]. Individuals may receive therapies against several allergens simultaneously and will be subject to parallel courses of such a dosing regimen (Table 1). Aluminium salts are the adjuvant of choice in both vaccinations and allergy immunotherapies because of their efficacy in promoting immune reactions to antigens (preventative therapy) and modifying the immune response to allergens (tolerance therapy) respectively. While the detailed mechanisms of their activities as adjuvants remain to be elucidated [4] their immunopotency is testimony to their biological availability and biological reactivity at injection sites and elsewhere in the body. Contrary to the view of a recent otherwise informed commentary [5] aluminium adjuvants are not ‘harmless salts’! They are far from being benign participants in vaccination and immunotherapy and their reactivity’s have been associated with adverse events in the recipients of such therapies. Adverse reactions to aluminium adjuvants might have been avoided if there had been a requirement to demonstrate the safety of their use in humans. However, no such regulations exist and the amount of aluminium salt which is included as an adjuvant is wholly determined by its immuno-efficacy in tandem with the respective antigen or allergen. Indeed it is an anomaly of many trials of the safety of aluminium-adjuvanted vaccines and immunotherapies that the (essentially toxic) aluminium adjuvant is considered to be the appropriate placebo in such clinical trials [6].

**Table 1:** Typical dosing regimens used in sub-cutaneous allergy immunotherapy

**Context**	**Criteria**	**Range**
First year	Range of number of injections for up dosing to a maintenance dose	4 to 16 injections [7-10]
	Range of number of injections for remaining first year maintenance course	4 to 12 injections [7-10]
Subsequent years	Range of number of injections for subsequent annual maintenance courses	6 to 12 injections [7-10]
	Recommended number of years of treatment	3 to 5 years [11,12]
Whole course	Range of number of injections for 3 years treatment for a single allergen	Up to 54 injections

### Adverse events associated with aluminium adjuvants in allergy immunotherapy

The safety of aluminium adjuvants used in vaccination is under increasing scrutiny and one serious disease, a neuromuscular disorder called macrophagic myofasciitis, is attributed to the persistence of aluminium salts at injections sites in muscle [13]. Established links with other more common conditions such as chronic fatigue syndrome [14] and autoimmune disease [15] are burgeoning though the incidences of adverse events remain a very small percentage of any vaccinated population. Herein it is already noted that in allergy immunotherapy not only the absolute number of injections of aluminium adjuvants but also their frequency, for example one a month or more (Table 1), often far exceeds that received through vaccinations. If the safety of aluminium adjuvants used in vaccinations is open to question then the safety of the same adjuvants used more frequently and in greater numbers in allergy immunotherapy must also be in question. While adverse reactions in individuals receiving allergy immunotherapy have been reported in the scientific and medical literature these reports are relatively few and far between [16,17]. Reactions include; foreign body granulomas [18], urticaria [19], sub-cutaneous sarcoidosis [20], progressive circumscribed sclerosis [21], sub-cutaneous nodules [22] and cutaneous-sub-cutaneous pseudolymphoma [23]. The actual incidence of adverse events such as the aforementioned is unknown and while the European Medicines Agency (EMA) lists as many as 32 adverse reactions to immunotherapy ranging from discolouration of the skin to encephalopathy their information cannot be verified as the data were not taken from an openly available peer-reviewed resource [24].

### Toxicity of aluminium adjuvants

To appreciate the safety issues which relate to the use of aluminium adjuvants in allergy immunotherapy we need an understanding of what happens to the injected aluminium salt. We need to consider the biological availability of aluminium both at the injection site and, as a consequence of the transport of both dissolved and particulate aluminium, beyond the immediate vicinity of the injection, for example in the lymph nodes. Details of the bioinorganic chemistry of aluminium adjuvants have been reviewed recently [4] and herein it is suffice to summarise such as the biological chemistry of the free aluminium cation, Al^3+^_(aq)_, which is the principal antagonist in aluminium toxicity [25]. Aluminium adjuvants, as sparingly soluble particulates, can be considered as sources of biologically available Al^3+^_(aq)_. How then could a sub-cutaneous source of biologically available Al^3+^_(aq)_ be responsible for adverse events or reactions in allergy immunotherapy?

#### Aluminium body burden

The, perhaps, simplest scenario would be that aluminium adjuvants contribute directly and significantly to an individual’s body burden of aluminium. The latter has recently been defined as the sum of aluminium atoms associated with the human body at any one moment in time [26]. Under this redefinition each injection of an aluminium adjuvant will contribute up to 1.25 mg of aluminium to the body burden of aluminium at the time of the injection. It is important to recognise that the body burden of aluminium is not evenly distributed throughout the body mass and that aluminium will be found as focal accumulations determined by the form of aluminium at exposure and its route of entry into the body [27]. By way of an example, evidence is beginning to demonstrate that aluminium administered as adjuvant may be phagocytosed as particulates by infiltrating cells and transported to distant and specific sites in the body including the lymph nodes but also into the brain [28]. However, contrary to burgeoning scientific evidence it is common practice by regulatory bodies, such as the European Food Standards Agency (EFSA) [29] or the previously mentioned EMA [24], to apply a safety criterion known as a tolerable weekly intake (TWI) to, almost universally, confirm the innocuous nature of all forms of human exposure to aluminium. This criterion, based wholly on the results of animal studies, assumes that all exposures to aluminium are ‘biochemically equal’ and that the route of exposure and the form of aluminium involved are unimportant. Nothing could be further from the truth where human exposure to aluminium is concerned [26]. A single injection of 1 mg of aluminium adjuvant will add 1 mg of aluminium to the body burden but this mg of aluminium will distribute throughout the body according to myriad different influences beginning with those occurring at the injection site [4]. Multiple injections of aluminium adjuvant over relatively short time periods, for example 15 injections over 12 months as might be common in allergy immunotherapies (Table 1), could result in significant focal accumulations of aluminium at single target sites and thereby present the possibility of aluminium toxicity at such sites. Allergy immunotherapy with aluminium adjuvants will contribute significantly to the aluminium body burden of the recipient and because of the nature of the route of exposure it is highly likely that the fate of a significant proportion of the adjuvant aluminium will be focal accumulations with the potential to exert a form of toxicity.

#### Aluminium the antigen

A second scenario whereby aluminium adjuvants in allergy immunotherapies might contribute towards adverse reactions to such therapies is related to the known antigenicity of aluminium [30]. It is perhaps less well appreciated that in addition to being an effective adjuvant it has also been shown that aluminium can act as an antigen. Thus, aluminium adjuvants in modifying the body’s immune response to allergens in immunotherapy may additionally sensitise the body to the presence of aluminium. The result is that following injection of an adjuvant-allergen therapy the body develops a memory of the exposure to aluminium. Subsequent multiple injections reinforce this memory and raises the possibility that the body is sensitised to not only the aluminium injected as adjuvant but potentially all body stores of aluminium. The body burden of aluminium becomes an immunological target and one which unfortunately the machinery of immunity is unable to deal with as it might ‘normal’ allergens/antigens. When aluminium the adjuvant is simultaneously aluminium the antigen there is the possibility that a relatively small exposure to aluminium, as might occur following a single injection of aluminium adjuvant in immunotherapy, could instigate a cascade of reactions whereby aluminium throughout the body is recruited to produce a significant immune/inflammatory-like response. The severity of such a response would depend upon both the body burden of aluminium and the location of significant deposits of aluminium. It is not yet clear which form or forms of aluminium are capable of acting as antigens [30]. If Al^3+^_(aq)_ is the antigen then this could help to explain why the body isn’t (presumably) on continuous immune alert against its everyday burden of aluminium. The concentration of Al^3+^_(aq)_ in the majority of physiological milieu will be nanomolar [27] and its propensity to act as an antigen will depend upon the kinetic inertia of deposits of aluminium in delivering Al^3+^_(aq)_ at a rate sufficient to fuel the formation of antibodies against it. Such situations could include sites of injection of aluminium adjuvants, the lymph nodes and known targets of aluminium in the brain including myelin.

#### Aluminium as a potent adjuvant

Another situation which might help to explain the putative toxicity of aluminium adjuvants in allergy immunotherapies and vaccinations is the outcome of the known potency of aluminium salts as adjuvants. Aluminium salts are not only effective in increasing the antigenicity of the allergens and antigens which are the targets of therapies they are also able to induce antigenicity in substances which would not otherwise act as antigens or allergens [31]. One example of this is to induce allergy to codfish by feeding mice with codfish and aluminium antacids [32]. Within a relatively short period of time the mice are demonstrating allergic responses to codfish alone. The potency of aluminium salts as adjuvants must be carefully considered in the preparation of allergy immunotherapies as any additional components or contaminants of the injections [33] could become antigenic and the target of an immune response both at the injection site and even beyond at other places in the body. The adjuvanticity of aluminium salts may also come into play in inducing antigenicity in constituents of foreign- or self-origin in the vicinity of the injection site. Actually such a mechanism may have a role to play in the efficacy of aluminium adjuvants in immunopotentiation though ideally these effects should not be translated elsewhere in the body where such would be unwanted and potentially toxic.

### Aluminium adjuvants in allergy immunotherapy need to be both effective and safe

Aluminium is an effective adjuvant in allergy immunotherapy because of its biological reactivity and that same reactivity means that there must be a number of mechanisms whereby aluminium adjuvants could also result in adverse events. One or more of these mechanisms could be a component of the, as yet non-elucidated, mode of action of aluminium adjuvants in stimulating or modifying immunity and in the majority of recipients of allergy therapies will result in little or limited discomfort. However, the likelihood is that each of these mechanisms will play some role, to a lesser or greater extent, in an individual’s response to the injection of aluminium adjuvant and in some individuals or particular circumstances the biological response to one or repeated injections of aluminium salt will be an adverse event. The difficulty is in predicting which individual or what set of circumstances is likely to instigate a biological cascade leading to toxicity. One probable predisposition would be an unusually high body burden of aluminium. There are many possible reasons why an individual’s body burden of aluminium is high including exposure to aluminium through extended periods of sub-cutaneous immunotherapy. Research is on-going into the best way of establishing by non-invasive means an individual’s body burden of aluminium and at present this is achieved by measuring urinary excretion of aluminium [14,26]. Future research should elaborate upon whether individuals with high body burdens of aluminium are more likely to suffer an adverse reaction to immunotherapy as well as whether individuals receiving extended periods of immunotherapy are more likely to have higher body burdens of aluminium and thereby predisposing themselves to an adverse event in the future.

## Conclusions

Aluminium hydroxide is currently the adjuvant of choice in sub-cutaneous allergy immunotherapy. Aluminium adjuvants are used to modify the immune response to a range of allergens and are generally used in multiple injections over extended time periods. Adverse events or reactions to allergy immunotherapy are documented and have been associated with aluminium adjuvants. While the mechanism of action of aluminium adjuvants in immunotherapy (likewise vaccination) has not been fully elucidated it will be allied to the biological reactivity of aluminium at both the injection site and elsewhere in the body. Adverse reactions to aluminium adjuvants may be amplifications of aluminium’s activity as an adjuvant and there may be subsets of individuals who are predisposed to such events. For example, individuals with a high body burden of aluminium, to which allergy immunotherapy will be a contributor, may be more susceptible to adverse events. There are few reliable data purporting to neither the safety nor the toxicity of aluminium adjuvants used in allergy immunotherapy. These gaps in knowledge should be remedied in future clinical trials of new immunotherapies and in records of clinical practice of therapies currently in use.

## Competing interests

CE has received reimbursement of travel and accommodation costs incurred through meetings with Allergy Therapeutics, a company which manufacturers immunotherapies which do not use aluminium adjuvants.

## Authors’ contributions

CE conceived the idea for the paper and wrote the manuscript.

## Authors’ information

CE is Professor of Bioinorganic Chemistry and Leader of the Research group on Aluminium and Silicon.
